# Optimization and pre-use suitability selection for wrist photoplethysmography-based heart rate monitoring in patients with cardiac disease

**DOI:** 10.1093/ehjdh/ztaf084

**Published:** 2025-07-23

**Authors:** Paulien Vermunicht, Christophe Buyck, Sebastiaan Naessens, Wendy Hens, Caro Verberckt, Emeline Van Craenenbroeck, Kris Laukens, Lien Desteghe, Hein Heidbuchel

**Affiliations:** Research Group Cardiovascular Diseases, University of Antwerp, Prinsstraat 13, Antwerp 2000, Belgium; Department of Cardiology, Antwerp University Hospital, Drie Eikenstraat 655 Antwerp 2650, Belgium; Research Group Cardiovascular Diseases, University of Antwerp, Prinsstraat 13, Antwerp 2000, Belgium; Department of Cardiology, Antwerp University Hospital, Drie Eikenstraat 655 Antwerp 2650, Belgium; Department of Cardiology, Antwerp University Hospital, Drie Eikenstraat 655 Antwerp 2650, Belgium; Department of Cardiology, Antwerp University Hospital, Drie Eikenstraat 655 Antwerp 2650, Belgium; Research Group Movant, University of Antwerp, Antwerp, Belgium; Department of Cardiology, Antwerp University Hospital, Drie Eikenstraat 655 Antwerp 2650, Belgium; Research Group Cardiovascular Diseases, University of Antwerp, Prinsstraat 13, Antwerp 2000, Belgium; Department of Cardiology, Antwerp University Hospital, Drie Eikenstraat 655 Antwerp 2650, Belgium; Department of Computer Science, University of Antwerp, Antwerp, Belgium; Biomedical Informatics Research Network Antwerp (Biomina), University of Antwerp, Antwerp, Belgium; Research Group Cardiovascular Diseases, University of Antwerp, Prinsstraat 13, Antwerp 2000, Belgium; Department of Cardiology, Antwerp University Hospital, Drie Eikenstraat 655 Antwerp 2650, Belgium; Faculty of Medicine and Life Sciences, Hasselt University, Hasselt, Belgium; Heart Center Hasselt, Jessa Hospital, Hasselt, Belgium; Department of Nursing and Midwifery Sciences, Centre for Research and Innovation in Care (CRIC), University of Antwerp, Antwerp, Belgium; Research Group Cardiovascular Diseases, University of Antwerp, Prinsstraat 13, Antwerp 2000, Belgium; Department of Cardiology, Antwerp University Hospital, Drie Eikenstraat 655 Antwerp 2650, Belgium; Faculty of Medicine and Life Sciences, Hasselt University, Hasselt, Belgium

**Keywords:** Exercise, Heart Rate, Wearable Electronic Devices, Fitness Trackers, Cardiac Rehabilitation

## Abstract

**Introduction:**

Sensor placement, activity type influencing wrist movements, and individual characteristics impact accuracy of wrist-worn photoplethysmography (PPG)-based heart rate (HR) monitors. This study investigated technical interventions to optimize PPG accuracy in patients with cardiac disease.

**Methods and results:**

The Fitbit Inspire 2 PPG monitor was evaluated across three cohorts, using a Polar H10 chest strap as reference: (ⅰ) 10 healthy volunteers performed wrist movements with the monitor placed one or three fingers above the wrist to identify optimal placement; (ⅱ) 10 volunteers engaged in sport activities (walking, running, cycling, rowing); (ⅲ) 30 cardiac rehabilitation patients were monitored during exercise to assess baseline accuracy. Patients with low accuracy [mean absolute percentage error (MAPE) < 10% for <70% of training time] underwent technical interventions (sensor cleaning, forearm shaving, position fixation, and/or relocation to the volar wrist). Placement three vs. one fingers above the wrist was significantly more accurate (mean difference in MAPE: −11.4%, *P* < 0.001). Walking showed the highest accuracy (MAPE = 3.8%), followed by cycling (MAPE = 6.9%) and running (MAPE = 8.5%), while rowing had the lowest accuracy (MAPE = 13.4%, *P* < 0.001). Among CR patients, 66.7% achieved high baseline accuracy. Technical interventions improved accuracy in 50.0% of those with low baseline accuracy, but no significant predictors of optimization success were identified.

**Conclusion:**

Accurate PPG-based monitoring requires a sensor placed higher on the wrist. Nevertheless, only two-thirds of patients are suitable for such monitoring, with improvement by technical adaptations possible (but impractical) in the others. Therefore, assessing baseline accuracy is a prerequisite before relying on these devices for activity guidance.

## Graphical Abstract

**Graphical Abstract:**
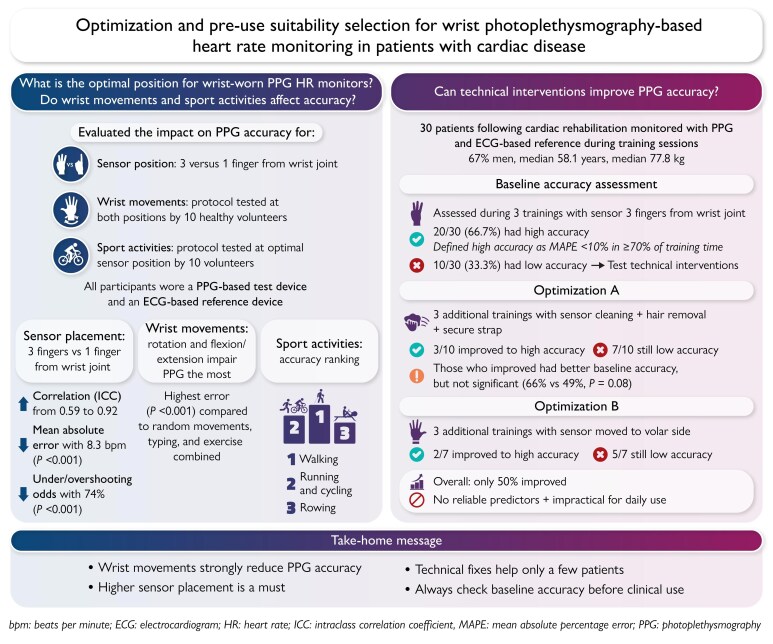

## Introduction

Wrist-worn photoplethysmography (PPG)-based heart rate (HR) monitors are increasingly popular tools for registering physical activity (PA) and providing training guidance.^1^ By detecting changes in blood volume through light absorption and reflection, PPG devices offer a non-invasive, convenient method for daily and long-term HR monitoring.^2^ Given the established link between physical inactivity and cardiovascular disease, these monitors have potential for tracking and enhancing PA in patients with cardiac disease.^3,4^

While previous studies have generally reported acceptable accuracy for wrist-worn PPG-based HR monitors,^5–8^ the reliability can vary due to multiple influencing factors.^9^ Firstly, individual characteristics beyond immediate control, such as age, sex, BMI, skin tone, and cardiovascular pathophysiology, may affect the optical properties of the skin and, therefore, the PPG signal.^10^ Arrhythmias like atrial fibrillation (AF) can introduce irregular pulse patterns, further impacting PPG accuracy.^6,7^ Moreover, erratic or cyclical wrist and arm movements can generate motion artefacts (MA) that disturb sensor-skin contact, distort light signals, reduce signal quality and cause HR over- or underestimations.^2^ Previous studies have shown that PPG accuracy decreases with higher-intensity activities involving more arm movement, finger gripping or wrist flexion/extension.^11–13^ However, most studies reported mean biases rather than absolute error metrics, which better reflect device accuracy because, mean biases can cancel out over- and underestimations, even when individual errors are large.^1^ Additionally, some wrist-worn HR monitors incorporate motion sensors to aid PPG-based HR detection, but the extent to which these corrections affect accuracy remains unclear due to the proprietary nature of device algorithms. Combined with the heterogeneity in results across devices, ongoing advancements in sensor technologies, algorithms and designs, this highlights the need for rigorous continued research to evaluate PPG accuracy during specific wrist movements and diverse physical activities, certainly if one wants to use these devices in clinical settings.^1,8^

Some factors that affect PPG accuracy are modifiable. Positioning the sensor further from the wrist joint may reduce sensitivity to MA due to less blood volume variation and interference from tendons and muscles, while maintaining a stable signal.^14^ Also, wearing the device on the volar side of the wrist, where vascular density is higher, fewer hair follicles are present, and anatomical interference from tendons and bones is lower, may provide a strong PPG signal.^15^ Despite some general recommendations from device manufacturers, such as Fitbit’s user manual, which suggests positioning the device on the dorsal side one finger from the wrist joint during rest and a bit higher during exercise, there is a lack of comprehensive research and formal guidelines on optimal sensor placement.^16^ Finally, technical aspects such as wristband tightness and sensor cleanliness, also influence signal quality.^17^ Maintaining consistent and tight sensor placement, potentially using band adjustments or support,^18^ and mitigating interference from body hair, debris and oil on the sensor could reduce PPG artefacts.^19^ Despite these factors’ potential impact, it remains unclear to what extent addressing these technical aspects can effectively improve PPG accuracy.

Therefore, this study aimed to evaluate the influence of sensor placement, wrist movements, exercise type, and technical adjustments on the measurement of HR by a wrist-worn PPG-based monitor in comparison with an electrocardiogram (ECG)-based chest strap.

## Methods

**Figure 1:**
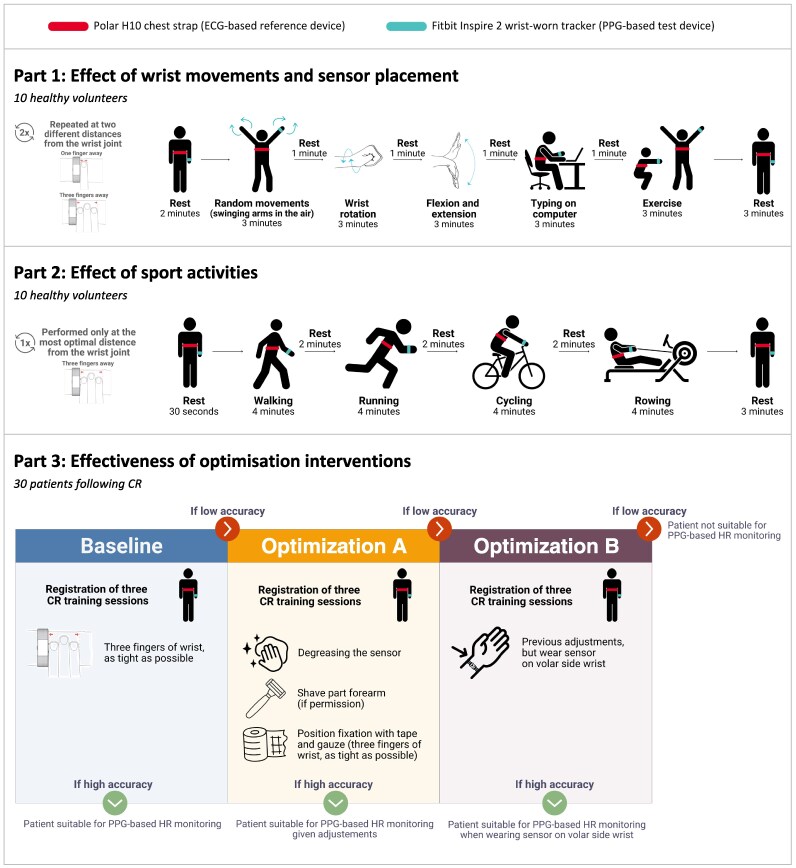
Methodology overview of the three research parts aiming to evaluate and optimize the accuracy of wrist photoplethysmography based heart rate monitoring. CR, cardiac rehabilitation; ECG, electrocardiogram; HR, heart rate; MAPE, mean absolute percentage error; PPG, photoplethysmography. High accuracy is defined as MAPE <10% for ≥70% of the training time. Low accuracy is defined as MAPE <10% for <70% of the training time.

This study comprises three distinct parts, each addressing a specific aspect of wrist-worn PPG-based HR monitoring accuracy and its optimization. An overview of the methodology is provided in *Figure 1*. Part 1 determines the optimal distance between sensor and wrist joint and explores the impact of wrist movements on PPG accuracy. Part 2 examines how different sports activities influence PPG performance. Part 3 evaluates PPG accuracy during exercise in patients following cardiac rehabilitation (CR) and investigates the potential for improving accuracy through technical interventions.

This study was conducted in accordance with ethical principles for medical research involving human subjects. Ethical approval for Part 3 was obtained from the Ethics Committee of Antwerp University Hospital (UZA) and University of Antwerp (UAntwerp), and all participants provided informed consent prior to participation.

### HR monitors

In all study parts, the Fitbit Inspire 2 wrist-worn tracker, which employs optical PPG technology, was used as test device, and the Polar H10 chest strap, which measures HR using electrical field signals, served as reference device. The Polar H10 was chosen due to its strong correlation with gold standard Holter measurements in prior validation studies.^20–23^ Before each measurement, the electrodes on the surface of the Polar chest strap were wetted to optimize conductivity, in agreement with manufacturer instructions. For the Fitbit PPG device, a single researcher ensured that the wristband was worn as tightly as possible while remaining comfortable.

### Part 1: effect of wrist movements and sensor placement

This part involved 10 healthy volunteers (≥18 years) performing a controlled protocol of specific wrist movements twice while wearing the Fitbit and Polar HR monitors (*Figure 1*, Part 1). In the first session, the Fitbit was placed at a one-finger-width distance from the wrist joint, and in the second session, the Fitbit was positioned at a three-fingers-width distance. This order was consistent for all participants. Both sessions were conducted on the same day, with a recovery period between sessions. In both sessions, the Fitbit was fitted by the same researcher aiming for consistent positioning.

The protocol consisted of five specific movements: random arm movements, wrist rotation, wrist flexion and extension, typing on a computer, and an exercise in which participants tapped their knees repeatedly and then stretched their arms upwards. Participants were instructed to maintain consistent movement speeds based on visual demonstration, ensuring comparable performance. Each movement lasted 3 min, with rest periods of 2 min before the protocol, 1 min between movements, and 3 min at the end.

### Part 2: effect of sport activities

In Part 2, 10 healthy volunteers performed four sport activities: walking, running, cycling and rowing (*Figure 1*, Part 2). Walking and running were performed on a treadmill, cycling on a stationary ergometer, and rowing on a rowing ergometer. Participants wore the Fitbit positioned at the distance identified as optimal in Part 1. Each activity was performed for 4 min, with rest periods of 30 s at the start, 2 min between activities, and 3 min at the end. Participants selected speeds and wattages that felt comfortable, which were retrospectively observed to range between 3 and 4.5 km/h for walking, 5.5–11 km/h for running, 80–150 W for cycling, and 75–115 W for rowing.

The durations of activities and recovery periods in Part 1 and Part 2 were standardized to ensure consistency and align with other PPG validation studies.^11^ These timeframes were considered sufficient to detect the chronotropic HR response and to assess whether the PPG device adequately tracked HR changes.

### Part 3: effectiveness of optimization interventions

This part investigated whether specific technical modifications could improve the accuracy of PPG-based HR monitoring in patients with cardiac disease exhibiting low accuracy during exercise (*Figure 1*, Part 3; Supplementary material online, *Figure S1*). A total of 30 patients participating in a CR programme were included to ensure sufficient representation of individuals with low PPG accuracy, allowing for a meaningful evaluation of optimization interventions based on expected variability in accuracy. Patients performed controlled exercise sessions as part of their CR, monitored with the Fitbit and Polar devices.

#### Participants and study design

Participants were recruited during the first 6 weeks of their CR programme. Inclusion criteria were age ≥18 years, having a smartphone, capacity to provide informed consent, and a history of prior myocardial infarction, percutaneous coronary intervention (PCI), cardiac ablation, or cardiac surgery. Exclusion criteria included severe heart failure (NYHA III-IV), inability to speak or read Dutch or English, and cognitive impairment.

Participants completed up to nine CR training sessions of one hour, consisting of a combination of strength exercises and moderate-to-high-intensity cardio training. Cardio exercises included treadmill walking and running, cycling, rowing, arm cycling, the cross-trainer, and the stairmaster. Strength exercises included chest press, leg press, low row, vertical traction, and the recumbent bike. The first three sessions served as baseline measurement under standard device guidance: the Fitbit was positioned three fingers above the wrist joint, tightened securely but comfortably, with positioning verified by the study team before each session. Baseline accuracy was assessed after these three sessions. Patients were classified as having high accuracy if the mean absolute percentage error (MAPE) was <10% during at least 70% of registered training time. The 10% MAPE threshold was based on previous research and the standard for HR monitors set by the American National Standards Institute (ANSI),^5,24,25^ while the 70% threshold was selected by our clinical team as a practical benchmark for reliable HR monitoring during exercise. Patients with low accuracy (MAPE <10% in <70% of training time) proceeded to the optimization phases (*Figure 1*, Part 3; Supplementary material online, *Figure S1*).

#### Optimization phases

Patients classified as having low accuracy at baseline entered the first optimization phase (optimization A). During this phase, three technical adjustments were applied to the Fitbit before each training session: (ⅰ) the sensor was cleaned and degreased to remove oils or dirt; (ⅱ) with participant consent, a part of the forearm was shaved to minimize interference from body hair; and (ⅲ) the Fitbit’s position on the wrist was fixated at three fingers above the wrist joint using tape and gauze to ensure consistent placement and reduce MA. After three training sessions with these adjustments, accuracy was reassessed, and measurements were classified as having high or low accuracy.

If accuracy remained low after optimization A, participants proceeded to a second optimization phase (optimization B). In this phase, the Fitbit was relocated from the wrist’s dorsal side to the volar side. The adjustments from optimization A (cleaning, shaving, and fixing the position) were maintained. After three additional training sessions, accuracy was reassessed.

During all training sessions, participants maintained a training diary to record each exercise's start and end times. This allowed for sub-analyses of the various exercise types performed during training. Rowing and arm cycling were categorized as intensive arm movements, whereas other strength and cardio exercises (e.g. treadmill walking, cycling, leg press, chest press) were classified as non-intensive arm movements.

### Data collection and processing

After monitoring, HR data from both devices were synchronized to pseudonymized accounts via the Fitbit and Polar Beat smartphone applications. The data were exported as CSV files: Polar data through the Polar Flow web service and Fitbit data via the Fitbit Web API. The HR data were received at 1-s intervals for Polar and at 5-s intervals for Fitbit.

### Statistical analysis

All statistical analyses were performed using SPSS Statistics version 29 (IBM Corp). All *P*-values were two-sided, and a significance level of *P* < 0.05 was used throughout. Normality of continuous variables was assessed using the Shapiro-Wilk test and visual inspection of histograms. When normality could not be assumed, non-parametric tests were selected accordingly.

Accuracy of the PPG device was evaluated using multiple statistical metrics. Reliability was assessed using the intraclass correlation coefficient (ICC), which quantifies the degree of agreement between repeated paired measurements. A two-way mixed-effects model with absolute agreement and single measures was used, as this approach is optimal for comparing two measurement methods (PPG and ECG) assessing the same construct (HR) across multiple individuals. Values closer to 1 indicate stronger agreement. Device error was quantified using the mean absolute error (MAE, in b.p.m.) and MAPE (MAPE, in %). Based on prior literature and ANSI standards, a MAPE <10% was considered clinically acceptable.^5,24,25^ Accurate training time was calculated as the proportion of datapoints with MAPE <10%. To classify error types, raw percentage error was also examined to distinguish undershooting (error ≤ −10%) and overshooting (error ≥ +10%).^5,24,25^

In Part 1 and 2 of the study, linear mixed models were used to compare MAE and MAPE across test conditions (e.g. sensor positions, movement types, sport activities). These models included participant as a random intercept to account for the correlation of repeated measures within individuals and included fixed effects for the condition of interest. Results were reported as estimated mean differences with corresponding 95% confidence intervals (CI). For binary outcomes such as the presence of under- or overshooting, generalized linear mixed models with a binary logistic link were applied, yielding odds ratios and 95% confidence intervals. For multiple group comparisons (e.g. different sport activities), pairwise comparisons were conducted using estimated marginal means with Bonferroni correction to adjust for multiple testing.

In Part 1, a Friedman test was used for comparing MAE and MAPE across all wrist movement types. Given the non-normal distribution, ordinal nature of the test conditions, and repeated measures within participants, the Friedman test was deemed suitable. In Part 3, accuracy was summarized per participant per session as one percentage score representing accurate training time, setting the statistical unit at the subject level. Comparisons between independent groups (e.g. high vs. low baseline accuracy) used Mann–Whitney *U* tests due to small sample sizes and non-normal distributions. Paired comparisons (e.g. baseline vs. post-optimization A or B) were analysed using Wilcoxon signed-rank tests. Differences in categorical variables (e.g. gender, medication use) were assessed using Fisher’s exact test, appropriate for small cell sizes.

Despite some non-normal distributions, descriptive statistics are reported as means and standard deviations when preferred for interpretability and consistency with clinical reporting practices. Statistical methods were selected based on data type, distribution, group structure, and repeated-measures considerations, ensuring appropriateness for each analysis across the study.

## Results

### Part 1: effect of wrist movements and sensor placement

The 10 participants consisted of 5 men and women each, with a median age of 24.5 years (interquartile range, IQR: 24.0–30.8) and median weight of 75.5 kg (IQR: 71.3–81.0). All participants wore the device on their non-dominant wrist. Nine participants had Fitzpatrick skin types I-II, and one had Fitzpatrick type 4.

**Figure 2:**
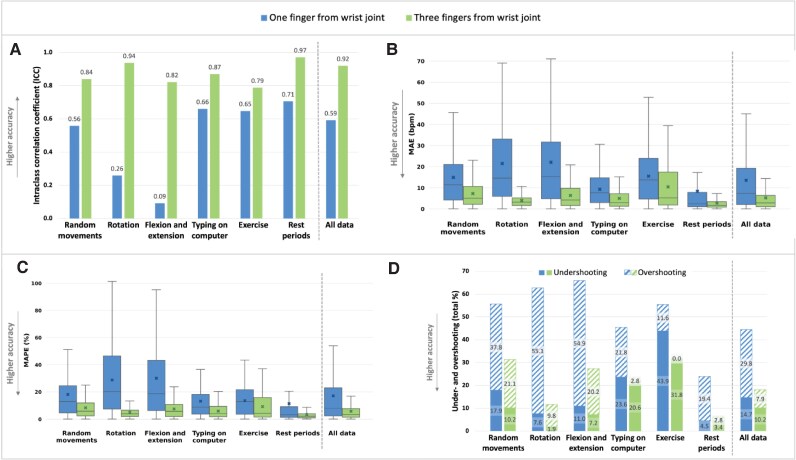
Accuracy metrics during various wrist movements with the PPG sensor placed one and three fingers from the wrist joint: (*A*) intraclass correlation coefficient, (*B*) MAE, (*C*) MAPE, and (*D*) total percentage of under- and overshooting. Bpm, beats per minute; ICC, intraclass correlation coefficient; MAE, mean absolute error; MAPE, mean absolute percentage error; PPG, photoplethysmography. Boxplots in panel B and C indicate the median, interquartile range (IQR), and whiskers extending to 1.5 × IQR; crosses represent the mean. Under/overshooting was defined as a percentage difference of 10% or more compared with the Polar device.

The placement of the PPG sensor three fingers above the wrist joint demonstrated highest accuracy (*Figure 2*, Supplementary material online, *Figure S2*). For all movement and rest periods combined, the three fingers distance resulted in a higher reliability (ICC = 0.92) compared with one finger (ICC = 0.59), a lower MAE (mean difference: −8.3 b.p.m., 95% CI: −8.8 to −7.8, *P* < 0.001), lower clinically acceptable MAPE (mean difference: −11.4%, 95% CI: −12.0 to −10.7, *P* < 0.001), and a lower likelihood of under- and overshooting (OR = 0.26, 95% CI: 0.24–0.29, *P* < 0.001).

During rest periods, the PPG device was more accurate compared with movement periods, for both sensor placements. For one-finger placement, the estimated MAPE was 20.5% during movement and 11.5% during rest (mean difference: −9.0%, 95% CI: −10.2 to −7.8, *P* < 0.001). Similarly, for three-finger placement, the MAPE was 7.3% during movement and 3.4% during rest (mean difference: −3.9%, 95% CI: −4.3 to −3.5, *P* < 0.001).

When comparing wrist movements at one finger distance, wrist rotation and flexion/extension showed the lowest accuracy, with significantly higher MAE (mean difference: 7.7 b.p.m., 95% CI: 6.5–8.9, *P* < 0.001) and MAPE (mean difference: 13.7%, 95% CI: 12.2–15.2, *P* < 0.001) compared with random movements, typing, and exercise combined. None of the movements met the clinical acceptability threshold (MAPE <10%). Even at three fingers distance, statistically significant differences between movements were observed (*P* < 0.001 for each metric), but these differences were less pronounced, with all movements showing clinically acceptable MAPE values (from 5.0% to 9.2%).

### Part 2: effect of sport activities

Of the 10 participants (7 women, 3 men), the median age was 23.5 years (IQR: 23.0–26.5) and median weight of 72.5 kg (IQR: 69.3–77.3). All participants wore the device on their non-dominant wrist. Nine participants had Fitzpatrick skin types I-II, and one had Fitzpatrick type 4.

**Figure 3:**
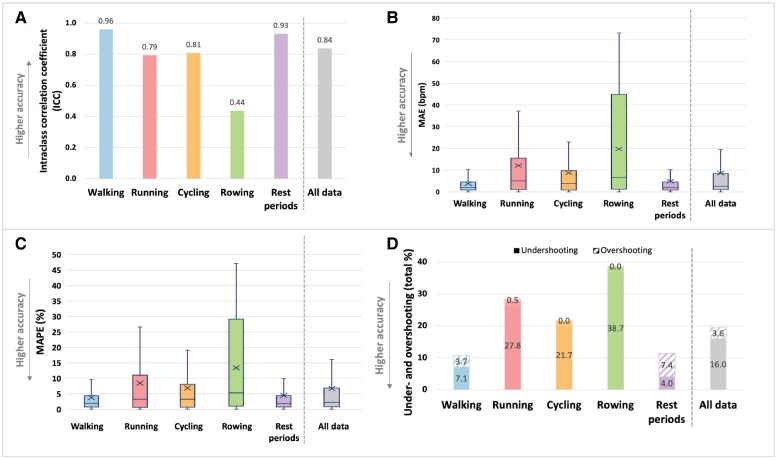
Accuracy metrics during various sport activities: (*A*) intraclass correlation coefficient (ICC), (*B*) MAE, (*C*) MAPE, and (*D*) total percentage of under- and overshooting. Bpm, beats per minute; ICC, intraclass correlation coefficient; MAE, mean absolute error; MAPE, mean absolute percentage error; PPG, photoplethysmography. Boxplots in panel B and C indicate the median, interquartile range (IQR), and whiskers extending to 1.5 × IQR; crosses represent the mean. Under/overshooting was defined as a percentage difference of 10% or more compared with the Polar device.

The PPG device positioned at three fingers above the wrist joint, demonstrated highest accuracy during walking and rest periods (*Figure 3*). Walking resulted in a very high reliability (ICC = 0.96), with the lowest MAE (3.8 b.p.m.) and MAPE (3.8%), while rest periods similarly showed very high reliability (ICC = 0.93) and low MAE (4.9 b.p.m.) and MAPE (4.5%). Differences in MAE and MAPE between walking and rest were not statistically significant (*P* = 0.31 for MAE, *P* = 0.37 for MAPE).

Running and cycling demonstrated moderate accuracy compared with walking and rest. Running had an ICC of 0.79, a MAE of 12.1 b.p.m. and a MAPE of 8.5%, with significantly higher values than walking (mean difference: 8.0 b.p.m. and 5.0%, both *P* < 0.001) and rest (mean difference: 6.8 b.p.m. and 3.7%, both *P* < 0.001). Cycling had an ICC of 0.81, a MAE of 8.7 b.p.m. and a MAPE of 6.9%, with significantly higher values than walking (mean difference: 4.7 b.p.m. and 3.0%, both *P* < 0.001) and rest (mean difference: 3.5 b.p.m. and 2.2%, both *P* < 0.001). Cycling showed slightly better accuracy than running, with significantly lower MAPE (mean difference: 1.5%, *P* = 0.017) and MAE (mean difference: 3.3 b.p.m., *P* < 0.001).

Rowing had the lowest accuracy of all activities, with an ICC of 0.44, the highest MAE (19.8 b.p.m.) and MAPE (13.4%). Both MAE and MAPE for rowing were significantly higher than for all other activities (all *P* < 0.001), with mean differences ranging from 7.1 to 15.1 b.p.m. for MAE and 4.6% to 9.8% for MAPE.

Visual analysis of PPG-based HR patterns (see Supplementary material online, *Figure S3*) showed that the PPG device often exhibited a lag in HR increase during the first minute of different activities. This was supported by artefact type analysis, which showed that undershooting was the predominant error type during all activities, with the highest undershooting time proportion observed during rowing (38.7%) and running (27.8%). Overshooting was minimal and only observed during walking (3.7%) and rest periods (7.4%).

### Part 3: effectiveness of optimization interventions

#### Baseline assessment (*n* = 30)

Baseline characteristics of the 30 patients with cardiac disease in Part 3, predominantly men (67%) with a median age of 58.1 years and a median weight of 77.8 kg, are summarized in *Table 1*. Although some patients had a history of AF, no episodes were reported or noted during the study period.

**Table 1:** Baseline characteristics of the study population in Part 3

	All patients (*n* = 30)	High accuracy at baseline (*n* = 20)	Low accuracy at baseline (*n* = 10)	*P*-value between high accuracy at baseline vs. low accuracy at baseline
**Age (years)**				**0**.**01**
Mean ± SD	58.1 ± 11.6	54.5 ± 10.6	65.3 ± 10.4	
Range	28–79	28–68	42–79	
**Gender**, Female, *n (%)*	10 (33.3)	7 (35.0)	3 (30.0)	1.00
**Weight (kg)**				**0**.**02**
Mean ± SD	77.8 ± 13.5	81.8 ± 12.1	69.8 ± 13.1	
Range	48.6–99.6	56.8–99.6	48.6–86.0	
**Height (cm)**				0.09
Mean ± SD	173.9 ± 12.4	176.3 ± 13.2	169.0 ± 9.3	
Range	148–209	155–209	148–184	
**BMI (kg/m²)**				0.18
Mean ± SD	25.8 ± 4.1	26.5 ± 4.2	24.3 ± 3.6	
Range	17.2–35.7	18.6–35.7	17.2–29.2	
**Skin color** ^a^, *n (%)*				0.54
Type 1	2 (6.7)	1 (5.0)	1 (10.0)	
Type 2	16 (53.3)	11 (55.0)	5 (50.0)	
Type 3	11 (36.7)	8 (40.0)	3 (30.0)	
Type 4	1 (3.3)	0 (0)	1 (10.0)	
Type 5 & 6	0 (0)	0 (0)	0 (0)	
**Hair density** ^b^, *n (%)*				0.29
Nil	4 (13.3)	2 (10.0)	2 (20.0)	
Sparse	15 (50.0)	12 (60.0)	3 (30.0)	
Moderate	11 (36.7)	6 (30.0)	5 (50.0)	
Dense	0 (0)	0 (0)	0 (0)	
**Indication CR**, *n (%)*				
PCI	14 (46.7)	10 (50.0)	4 (40.0)	0.71
Cardiac ablation	6 (20.0)	5 (25.0)	1 (10.0)	0.63
Myocardial infarction	2 (6.7)	1 (5.0)	1 (10.0)	1.00
Cardiac surgery	8 (26.7)	4 (20.0)	4 (40.0)	0.38
**Medical history**, *n (%)*				
Atrial fibrillation	7 (23.3)	5 (25.0)	2 (20.0)	1.00
Heart failure	4 (13.3)	3 (15.0)	1 (10.0)	1.00
Coronary artery disease	22 (73.3)	14 (70.0)	8 (80.0)	1.00
CVA/TIA	3 (10.0)	2 (10.0)	1 (10.0)	1.00
Myocardial infarction	7 (23.3)	5 (25.0)	2 (20.0)	1.00
Valve disease	4 (13.3)	2 (10.0)	2 (20.0)	0.58
Vascular disease	4 (13.3)	2 (10.0)	2 (20.0)	0.58
**Cardiovascular risk factors**, *n (%)*				
Hypercholesteremia	26 (86.7)	18 (90.0)	8 (80.0)	0.58
Hypertension	11 (36.7)	7 (35.0)	4 (40.0)	1.00
Diabetes mellitus	2 (10.0)	2 (10.0)	0 (0.0)	0.54
Smoking status				0.72
*Previous*	10 (33.3)	7 (35.0)	3 (30.0)	
*Current*	1 (3.3)	1 (5.0)	0 (0.0)	
*Never*	19 (63.3)	12 (60.0)	7 (70.0)	
**Medication use**, *n (%)*				
Rate control	22 (73.3)	15 (50.0)	7 (70.0)	1.00
*Beta blocker*	18 (60.0)	12 (60.0)	6 (60.0)	1.00
*Calcium antagonist*	4 (13.3)	3 (15.0)	1 (10.0)	1.00
Rhythm control	6 (20.0)	4 (20.0)	2 (20.0)	1.00

Bold *P*-values are significant.

BMI, body mass index; CR, cardiac rehabilitation; CVA, cerebrovascular accident; TIA, transient ischemic attack.

^**a**^Skin type was determined according to the Fitzpatrick classification, ranging from skin type 1 (pale white skin) to type 6 (dark brown or black skin).^26^

^**b**^Hair density of the forearm was graded into four categories by comparing the forearm of the participant to a set of previously described set of photographs, ranging from nil to dense.^27^ ‘Indication CR’ refers to the primary reason for enrolling in the cardiac rehabilitation programme at the time of the study, while ‘Medical history’ includes all relevant cardiovascular conditions present in the patient’s medical background.

**Figure 4:**
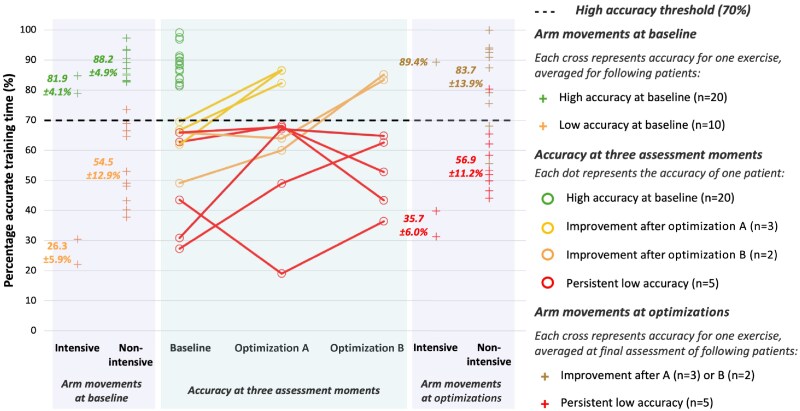
Percentage of accurate training time (MAPE <10%) per participant, per assessment moment, and split for arm movement intensity. MAPE, mean absolute percentage error. High accuracy is defined as MAPE <10% for ≥70% of training time (everything above the black threshold dotted line). Low accuracy is defined as MAPE <10% for <70% of training time (everything below the black threshold dotted line). Following exercises are classified as intensive arm movements: arm bike, rowing machine. Following exercises are classified as non-intensive arm movements: strength training (including leg press, chest press, low row, vertical traction, recumbent bike), cross-trainer, stairmaster, bike, walking/running on treadmill.

Of the total cohort, 20 patients (66.7%) demonstrated high baseline PPG accuracy, achieving a mean of 88.4 ± 5.2% accurate training time (i.e. data with MAPE <10%, *Figure 4*). This was significantly higher than the remaining 10 patients (33.3%), who had an average of 54.3 ± 15.7% accurate training time despite wearing the Fitbit at 3 fingers above the joint (*P* < 0.001). Compared with the high-accuracy group, the low-accuracy group was significantly older (65.3 years vs. 54.5 years, *P* = 0.01) and had a lower weight (69.8 kg vs. 81.8 kg, *P* = 0.02). No significant differences were observed for other baseline characteristics (*Table 1*).

**Figure 5:**
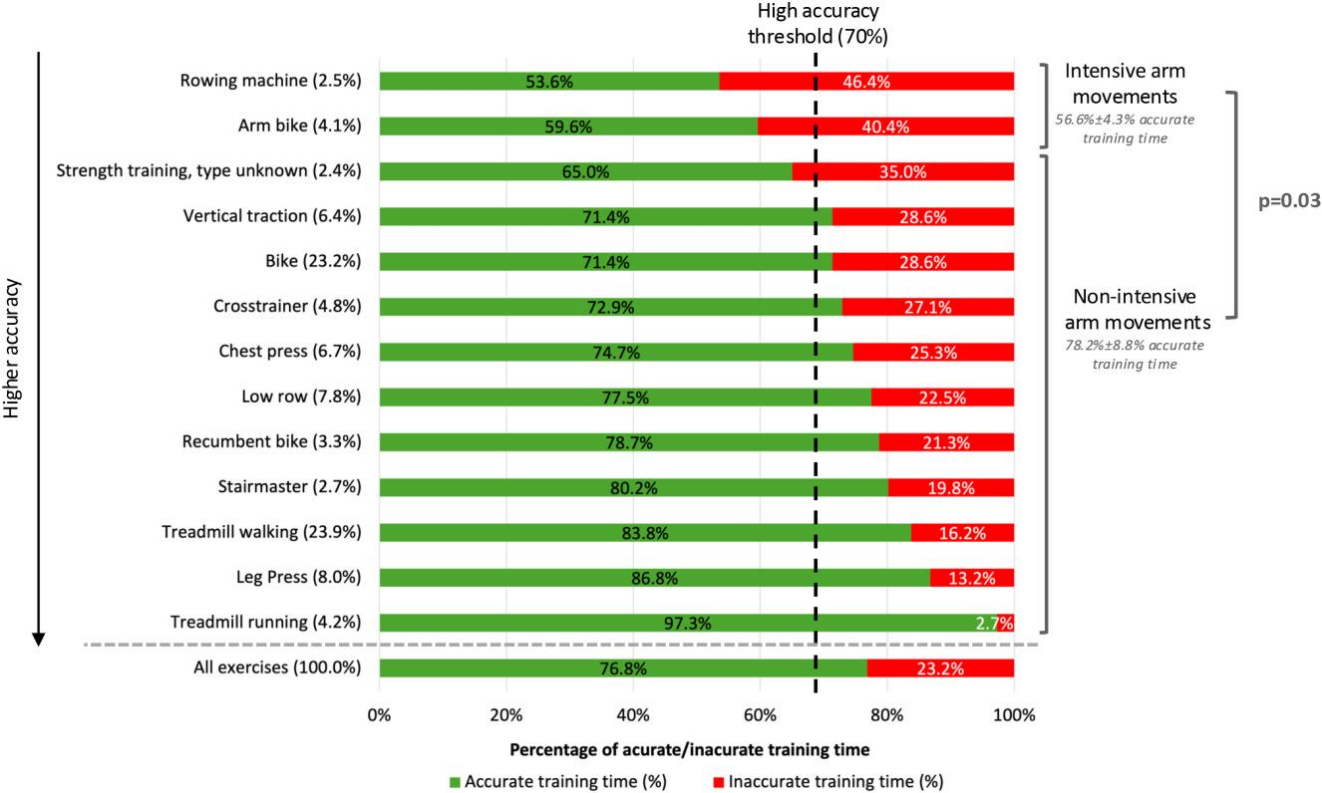
Percentage of accurate training time (MAPE <10%) by exercise type for baseline assessment (*n* = 30). MAPE: mean absolute percentage error. Results are ranked from the lowest to the highest accuracy.

Sub-analyses of exercise types performed during baseline training sessions of all patients showed that activities involving intensive arm movements, i.e. rowing and arm biking, were associated with a significantly lower percentage of accurate PPG measurements (56.6 ± 4.3%) compared with exercises involving non-intensive arm movements (78.2 ± 8.8%; *P* = 0.03) (*Figure 5*). When divided by baseline accuracy, the high baseline accuracy group maintained high accuracy (>70%) across all activities, including those with intensive arm movements (81.9 ± 4.1% vs. 88.2 ± 4.9% for intensive vs. non-intensive, *P* = 0.11). In the low baseline accuracy group, intensive arm movements were significantly less accurate (26.3 ± 5.9%) than non-intensive arm movements (54.5 ± 12.9%, *P* = 0.03) (see Supplementary material online, *Figure S4*).

#### Optimization A (*n* = 10)

After applying optimization A (i.e. sensor cleaning, shaving, and position fixation), three of the 10 patients (30%) with low baseline accuracy improved above the threshold for high accuracy (*Figure 4*). This subgroup demonstrated an average increase of 19.1 ± 4.8% in accurate training time, achieving a mean of 85.2 ± 5.4% post-optimization A (baseline vs. post-optimization A: *P* = 0.11). The remaining seven patients (70%) retained low accuracy, with no significant improvement in training data accuracy (mean accurate training time of 56.4 ± 17.8% post-optimization A; baseline vs. post-optimization A: *P* = 0.31). Notably, patients who improved after optimization A had a higher baseline percentage of accurate training time (66.0 ± 3.8%) compared with those who did not (49.3 ± 16.3%), although this difference was not statistically significant, likely due to the small groups (*P* = 0.08).

#### Optimization B (*n* = 7)

Of the seven patients with remaining low accuracy despite optimization A, two (28.6%) improved to high accuracy after optimization B (i.e. sensor relocation to the volar wrist side), with a mean increase of 26.8 ± 13.2% in accurate training time (57.5 ± 11.9% at baseline vs. 84.3 ± 1.3% post-optimization B: *P* = 0.18, *Figure 4*). The remaining five patients (71.4%) showed no significant improvement, with an average increase of only 5.9 ± 26.6% (47.1 ± 17.7% at baseline vs. 52.0 ± 12.2% post-optimization B: *P* = 0.67).

Overall, across both optimizations, five of the original 10 patients with low baseline accuracy (50%) achieved high accuracy. No significant differences in baseline characteristics, including age (*P* = 0.21), weight (*P* = 0.92), BMI (*P* = 0.92), baseline accuracy (*P* = 0.12), or gender (*P* = 1.00), were found between patients who improved and those who did not, although admittedly, the groups are small.

## Discussion

This study confirmed the significant influence of sensor placement, wrist movements, and type of sport activities on the accuracy of a wrist-worn PPG-based HR monitor. Additionally, it demonstrated the limited potential of technical interventions to optimize PPG accuracy during CR training sessions. Our findings suggest that some patients may not be suitable for PPG-based HR monitoring.

### Placement of the PPG sensor higher on the wrist for optimal accuracy

In Part 1, we demonstrated that positioning the PPG sensor further from the wrist joint (i.e. at a distance of three fingers) significantly improved accuracy, with a lower MAPE (mean difference: −11.4%, 95% CI: −12.0 to −10.7, *P* < 0.001) compared with placement near the wrist joint. We have demonstrated that dynamic wrist movements, such as rotation and flexion/extension, adversely affect accuracy (mean difference in MAPE: 13.7%, 95% CI: 12.2–15.2, *P* < 0.001). The higher sensor placement likely minimizes the impact of such wrist movements due to reduced sensor shifting relative to the skin and decreased deformation of the tissue under the sensor.^28^ This placement also reduces interference from tendons and bones while providing more stable blood flow in the monitored area, collectively mitigating MA and enhancing signal stability during exercises.^14^ Therefore, such position is a must for PPG-based HR monitoring (and was hence used in the other parts of our study).

Our findings align with previous research identifying wrist movements as a primary source of PPG signal distortion.^28^ Studies have also suggested that PPG signals obtained from less peripheral sites, such as the upper arm or forearm, exhibit fewer artefacts compared with those from the wrist region.^14^ However, our study is the first to directly compare sensor placements on the wrist itself (i.e. one finger vs. three fingers from the wrist joint), a site that remains practical for daily use. This novel insight underscores that ensuring proper placement is a simple and impactful step to enhance the reliability of wrist-worn PPG devices.

### Reduced PPG accuracy during activities involving intense arm movement

In Part 2, we demonstrated that the accuracy of the PPG device varied significantly across different exercise types. Although the device the Fitbit Inspire 2 HR monitor itself has not yet been validated in literature, other Fitbit models have been assessed in similar studies, and these data were used as a basis for comparison. Activities involving limited arm movements, such as walking, yielded the highest accuracy (MAE = 3.8 b.p.m., MAPE = 3.8%). Our results were slightly better than those reported in previous studies, such as Nelson *et al.*,^5^ who found a MAE of 9.5 b.p.m.,^5^ and Etiwy *et al.*,^23^ who reported a MAPE of 8.6% during walking.^23^ Our study observed moderate accuracy during cycling (MAE = 8.7 b.p.m., MAPE = 6.9%) and running (MAE = 12.1 b.p.m., MAPE = 8.5%), possibly influenced by the gripping of handlebars during cycling and MA generated by repetitive arm movements during running. For cycling on a stationary ergometer, reported MAPE values in the literature range from 8.4% to 21.1%,^9,11,13,23,29^ but are all above our observed 6.9%, while for treadmill running, reported MAE and MAPE values are 3.1 b.p.m. and 6.3%, respectively.^9,13^ These differences may reflect variations in study methodologies (e.g. healthy volunteers vs. patients with cardiac disease, continuous measurements vs. selected timepoints), device-specific algorithms, and individual factors such as gripping patterns and arm movement dynamics. Our better findings during walking and cycling may certainly be related to the 3-finger position as discussed above. With our accuracy, HR measuring during the most common fitness activities is reassuring.

Our study was the first to report PPG accuracy during rowing, and it was significantly lower (MAE = 19.8 b.p.m., MAPE = 13.4%) compared with the other activities, which may be attributed to repetitive and forceful wrist flexion and gripping motions. Although specific studies on rowing are lacking, Gillinov *et al*.^11^ reported a MAPE of 11.7% for cross-trainer use, an activity also involving upper-limb motion.^11^ The slightly higher accuracy during cross-trainer use may reflect it’s more fluid and less forceful arm movements compared with rowing. Similarly, in Part 3, sub-analyses of specific exercise types performed during baseline CR training sessions showed that the activities involving intensive arm movements, such as rowing and arm biking, were associated with significantly lower accuracy (56.6% ± 4.3% accurate training time) compared with non-intensive arm movements like treadmill walking and leg press exercises (78.2% ± 8.8%, *P* = 0.03). However, in the high-accuracy group, this observation was less pronounced and not significant, as all activities maintained reliable accuracy (>70%), including rowing (78.9%) and arm biking (84.8%). These findings collectively indicate that activities destabilising sensor placement or inducing substantial wrist motion consistently amplify MA and degrade PPG signal quality, whether during controlled experiments or real-world CR training sessions, but particularly in patients with low baseline accuracy.

Furthermore, during sport activities in Part 2, we observed predominantly undershooting errors, contrasting with the overshooting tendency observed during wrist movements in Part 1. This aligns with the observation of a lag in HR increase detection by PPG sensors at the onset of activities, supported by findings from other studies.^13,30,31^ This lag can be attributed to physiological factors such as reduced peripheral resistance and changes in pulse pressure during exercise, which temporarily affect the detection of blood flow by PPG sensors.^32^ While PPG accuracy tends to improve as exercise progresses and HR increases,^13^ the initial lag poses implications as it may lead to underestimated PA intensity during shorter bouts of exercise when these monitors are used for PA monitoring and guidance.

### Preselection of patients is necessary for reliable PPG monitoring

When positioning the PPG monitor three fingers above the wrist joint and ensuring a snug fit, our study found in Part 3 that 66.7% of patients with cardiac disease achieved high baseline accuracy during CR training sessions, defined as at least 70% of training time with a MAPE <10%, including during intensive arm movements such as rowing and arm biking. This high-accuracy group was significantly younger compared with the low-accuracy group, consistent with prior research showing that increased arterial stiffness and changes in skin characteristics with age can degrade PPG signal quality.^24^ Less expectedly, the high-accuracy group had a significantly higher weight, while height and BMI were also higher, albeit not statistically significant. Since a higher BMI is generally associated with lower PPG signal quality due to increased skin thickness and altered blood flow dynamics,^10^ our findings do not indicate a clear trend supporting or contradicting this expectation. Blok *et al*.^19^ also observed deviations from this general trend, associating a higher BMI with better PPG accuracy, though the underlying reasons remain unclear.^19^ These inconsistencies underscore the complex and sometimes contradictory interplay of physical characteristics influencing PPG performance, highlighting the difficulty of predicting PPG accuracy based on individual factors.

Our study was the first to evaluate several technical interventions aimed at improving PPG performance in patients with low baseline accuracy. Optimization A (sensor cleaning, shaving, and wrist position fixation) led to high accuracy in 30% of these patients, while a further 20% improved following optimization B (relocation to the volar wrist side). Despite these successes, 50% did not experience meaningful improvements, maintaining low accuracy levels post-optimization.

Patients who responded positively to optimization A tended to have higher baseline accuracy compared with non-responders (66.0% vs. 49.3%, *P* = 0.08), suggesting that baseline performance may influence responsiveness to technical adjustments. However, this association was not statistically significant, and no other baseline characteristics, including age, weight, or gender, were identified as predictors of improvement. This inability to reliably predict which patients will benefit from optimization strategies, combined with the overall low response rate, and the impracticality of doing this on a daily basis, leads us to conclude that, beyond positioning the monitor correctly on the wrist, as demonstrated in Part 1, additional technical interventions are of limited practical value for patients with low baseline accuracy. I.e. some patients are ‘not made for PPG-based HR monitoring’…

Hence, our findings indicate the importance of assessing baseline PPG accuracy (i.e. PPG-compatibility) before relying on these monitors for HR-based PA-load prediction and guidance. Early identification of patients with inherently poor PPG performance is essential, as alternative HR monitoring strategies, such as ECG-based chest straps, may be required to ensure accurate and reliable PA guidance, despite their reduced convenience for continuous monitoring.^11,33^ This can be achieved by performing a brief compatibility assessment prior to clinical use, consisting of three training sessions during which HR is simultaneously recorded with the PPG device and a reference ECG-based device, and subsequently calculating the proportion of accurate measurements (MAPE <10%), with ≥70% accurate training time serving as a practical threshold to define PPG compatibility. This approach offers a novel pragmatic tool to support appropriate patient selection when considering PPG-based HR monitoring with consumer-grade devices.

### Study strengths and limitations

This study provided valuable insights into the factors influencing the accuracy of wrist-worn PPG-based HR monitors, but some limitations should be acknowledged when interpreting the findings. Despite the relatively small sample sizes in Parts 1 and 2, and the subgroup undergoing optimization in Part 3, the combination led to valuable insights. Larger studies may further validate and extend these observations, particularly regarding potential predictors of baseline accuracy and responsiveness to optimization strategies.

While the measurement order in Part 1 was not randomized, fatigue effects were unlikely as intense movements occurred last, following low-intensity tasks, with sufficient rest before repeating the protocol. Similarly, in Part 2, the fixed order (walking, running, cycling, rowing) was unlikely to bias results, as all participants were well-trained and received adequate recovery time between activities.

Our study focused on a single PPG device, the Fitbit Inspire 2, which may limit the generalizability of our findings to other devices with different algorithms and sensor technologies. Also, the Fitbit device operates as a proprietary system, meaning that the exact algorithm used for HR estimation remains unknown. While the device possibly incorporates accelerometer data to mitigate motion artefacts, we cannot determine the extent of its influence on HR accuracy.

Moreover, commercially available wrist-worn devices such as Fitbit do not provide access to raw PPG waveform signals or continuous accelerometer data. This prevents independent evaluation of both signal quality and motion-induced errors based on accelerometery. HR data were received in 5-s intervals during the recorded activities in this study, but under other conditions, sampling rates may vary (e.g. every 10, 15 or 20 s) and it remains unclear whether these variations occur dynamically, for example, to conserve battery power or in response to motion. We fully acknowledge that alternative approaches, as demonstrated in recent studies by Reiss *et al*.^34^ and Meier *et al*.^34,35^ allow comprehensive access to raw PPG waveforms and IMU data using specialized research-grade equipment or custom-built sensors. These studies provide highly valuable contributions to algorithm development and technical signal processing optimization. However, our present study aimed to evaluate the clinical feasibility and optimization options of currently available commercial PPG devices as used by patients and clinicians in real-world ambulatory settings, where raw signal access is not provided. Our findings therefore directly inform the practical clinical implementation context, complementing technical signal processing studies.

While this ‘black box’ nature introduces uncertainty, we selected the Fitbit device due to its ability to provide continuous 24-h HR data export via the Fitbit Web API, an essential requirement for our overarching research objective of assessing continuous HR-based PA intensity in patients with cardiac disease. Nevertheless, our findings highlight the need for greater transparency from device manufacturers to enable more rigorous validation of PPG-based HR monitoring in clinical and research settings.

Certain factors known to influence PPG signal quality were not systematically measured. For example, although wristband tightness was standardized subjectively by trained investigators, it was not objectively measured using pressure sensors. The device was secured using a standard wristband with discrete adjustment holes rather than an elastic strap. Although this may have introduced some variability, it is unlikely to have systematically biased our findings, especially given that in Part 3, tightening the strap with gauze did not significantly improve accuracy. Additionally, factors such as grip pressure during cycling and rowing, arm movement patterns during walking and running, and environmental variables (e.g. ambient light and temperature) were not quantified. While these variables were controlled as much as possible across sessions, e.g. measurements were conducted in a climate-controlled hospital exercise room with stable temperature and humidity, individual physiological responses such as sweating and microvascular circulation could still contribute to variability in PPG accuracy. Since such individual differences also influence PPG performance in real-world conditions, this highlights the importance of assessing baseline accuracy before clinical implementation. Finally, the controlled exercise protocols used in Parts 2 and 3 may not fully capture the variability and complexity of real-world activities. Future studies could explore the impact of such real-world conditions to complement our findings.

## Conclusions

This study shows that the accuracy of PPG-based HR monitoring is influenced by both sensor placement and activity type, but more importantly, that there are compatible and incompatible patients for PPG-monitoring. Positioning the sensor higher on the wrist enhances accuracy and is a must for accurate PPG-based HR monitoring. Activities involving intensive arm movements substantially impair performance. In initially incompatible patients, interventions such as sensor cleaning, fixation, and/or volar positioning can lead to improvement in approximately half of cases, but this is not feasible on a daily basis. Therefore, assessing baseline accuracy is a prerequisite before relying on these devices for activity guidance.

Future studies may further validate these findings in larger and more diverse patient populations. Additionally, they could explore whether specific patient characteristics can reliably predict PPG compatibility, potentially allowing targeted preselection for PPG-based monitoring, complemented by an objective baseline compatibility check using a reference device before clinical application. Long-term real-world studies are also needed to assess performance and influencing factors during free-living conditions and daily-life activities, to optimize PPG-based ambulatory activity guidance in patients with cardiac disease.

## Supplementary Material

ztaf084_Supplementary_Data

## Data Availability

Raw data supporting the conclusions of this article will be made available by the authors upon request.

## Lead author biography

Paulien Vermunicht graduated as a Biomedical Scientist from the University of Antwerp, Belgium. She is currently pursuing a PhD under the supervision of Prof Dr Hein Heidbuchel and Prof Lien Desteghe, affiliated with the University of Antwerp and Antwerp University Hospital. Her research focuses on digital health innovations for patients with cardiovascular diseases, specifically the use of wearable heart rate monitors and algorithms to enhance the monitoring and management of PA in this patient population.

## References

[1] Zhang Y, Weaver RG, Armstrong B, Burkart S, Zhang S, Beets MW, et al Validity of wrist-worn photoplethysmography devices to measure heart rate: a systematic review and meta-analysis. J Sports Sci 2020;38:2021–2034. 32552580 10.1080/02640414.2020.1767348

[2] Scardulla F, Cosoli G, Spinsante S, Poli A, Iadarola G, Pernice R, et al Photoplethysmograhic sensors, potential and limitations: is it time for regulation? A comprehensive review. Measurement 2023;218:113150.

[3] Cleven L, Krell-Roesch J, Nigg CR, Woll A. The association between physical activity with incident obesity, coronary heart disease, diabetes and hypertension in adults: a systematic review of longitudinal studies published after 2012. Bmc Public Health 2020;20:726–726. 32429951 10.1186/s12889-020-08715-4 PMC7238737

[4] Dempsey PC, Rowlands AV, Strain T, Zaccardi F, Dawkins N, Razieh C, et al Physical activity volume, intensity, and incident cardiovascular disease. Eur Heart J 2022;43:4789–4800. 36302445 10.1093/eurheartj/ehac613

[5] Nelson BW, Allen NB. Accuracy of consumer wearable heart rate measurement during an ecologically valid 24-hour period: intraindividual validation study. JMIR Mhealth Uhealth 2019;7:e10828. 30855232 10.2196/10828 PMC6431828

[6] Al-Kaisey AM, Koshy AN, Ha FJ, Spencer R, Toner L, Sajeev JK, et al Accuracy of wrist-worn heart rate monitors for rate control assessment in atrial fibrillation. Int J Cardiol 2020;300:161–164. 31787389 10.1016/j.ijcard.2019.11.120

[7] Quinn R, Leader N, Lebovic G, Chow C-M, Dorian P. Accuracy of wearable heart rate monitors during exercise in Sinus rhythm and atrial fibrillation. J Am Coll Cardiol 2024;83:1177–1179. 38508851 10.1016/j.jacc.2024.01.024

[8] Ibrahim NS, Rampal S, Lee WL, Pek EW, Suhaimi A. Evaluation of wrist-worn photoplethysmography trackers with an electrocardiogram in patients with ischemic heart disease: a validation study. Cardiovasc Eng Technol 2024;15:12–21. 37973701 10.1007/s13239-023-00693-z

[9] Muggeridge DJ, Hickson K, Davies AV, Giggins OM, Megson IL, Gorely T, et al Measurement of heart rate using the polar OH1 and fitbit charge 3 wearable devices in healthy adults during light, moderate, vigorous, and sprint-based exercise: validation study. JMIR Mhealth Uhealth 2021;9:e25313. 33764310 10.2196/25313 PMC8088863

[10] Fine J, Branan KL, Rodriguez AJ, Boonya-ananta T, Ajmal, Ramella-Roman JC, et al Sources of inaccuracy in photoplethysmography for continuous cardiovascular monitoring. Biosensors (Basel) 2021;11:126 33923469 10.3390/bios11040126 PMC8073123

[11] Gillinov S, Etiwy M, Wang R, Blackburn G, Phelan D, Gillinov A, et al Variable accuracy of wearable heart rate monitors during aerobic exercise. Med Sci Sports Exerc 2017;49:1697–1703. 28709155 10.1249/MSS.0000000000001284

[12] Hermand E, Cassirame J, Ennequin G, Hue O. Validation of a photoplethysmographic heart rate monitor: polar OH1. Int J Sports Med 2019;40:462–467. 31189190 10.1055/a-0875-4033

[13] Horton JF, Stergiou P, Fung TS, Katz L. Comparison of polar M600 optical heart rate and ECG heart rate during exercise. Med Sci Sports Exerc 2017;49:2600–2607. 29135785 10.1249/MSS.0000000000001388

[14] Maeda Y, Sekine M, Tamura T. Relationship between measurement site and motion artifacts in wearable reflected photoplethysmography. J Med Syst 2011;35:969–976. 20703691 10.1007/s10916-010-9505-0

[15] Matsui Y . ‘Introduction to Kienböck’s Disease’; Chapter: ‘Wrist Anatomy and Vascularity’. Singapore: Springer; 2023.

[16] Fitbit. Fitbit Inspire 2 User Manual, Version 1.0. 2020. https://www.fitbit.com/content/assets/help/manuals/manual_inspire_2_en_US.pdf.

[17] Bretonneau Q, Peruque-Gayou E, Wolfs E, Bosquet L. Parameters influencing the accuracy of a wrist photoplethysmography heart-rate monitor (polar unite) during exercise. Int J Sports Physiol Perform 2023;18:440–443. 36805933 10.1123/ijspp.2022-0288

[18] Sartor F, Papini G, Cox LGE, Cleland J. Methodological shortcomings of wrist-worn heart rate monitors validations. J Med Internet Res 2018;20:e10108. 29967000 10.2196/10108 PMC6048383

[19] Blok S, Piek MA, Tulevski II, Somsen GA, Winter MM. The accuracy of heartbeat detection using photoplethysmography technology in cardiac patients. J Electrocardiol 2021;67:148–157. 34256184 10.1016/j.jelectrocard.2021.06.009

[20] Vermunicht P, Makayed K, Meysman P, Laukens K, Knaepen L, Vervoort Y, et al Validation of polar H10 chest strap and fitbit inspire 2 tracker for measuring continuous heart rate in cardiac patients: impact of artefact removal algorithm. Europace 2023;25:euad122.550–euad122.550.

[21] Schaffarczyk M, Rogers B, Reer R, Gronwald T. Validity of the polar H10 sensor for heart rate variability analysis during resting state and incremental exercise in recreational men and women. Sensors (Basel) 2022;22:6536. 36081005 10.3390/s22176536 PMC9459793

[22] Merrigan JJ, Stovall JH, Stone JD, Stephenson M, Finomore VS, Hagen JA, et al Validation of garmin and polar devices for continuous heart rate monitoring during common training movements in tactical populations. Meas Phys Educ Exerc 2023;27:234–247.

[23] Etiwy M, Akhrass Z, Gillinov L, Alashi A, Wang R, Blackburn G, et al Accuracy of wearable heart rate monitors in cardiac rehabilitation. Cardiovasc Diagn The 2019;9:262–271. 10.21037/cdt.2019.04.08 PMC6603497 31275816

[24] Chow HW, Yang CC. Accuracy of optical heart rate sensing technology in wearable fitness trackers for young and older adults: validation and comparison study. JMIR Mhealth Uhealth 2020;8:e14707. 32343255 10.2196/14707 PMC7218601

[25] Association for the Advancement of Medical Instrumentation and American National Standards Institute . Cardiac Monitors, Heart Rate Meters, and Alarms. Volume 2.2. Arlington, VA: AAMI; 1995.

[26] Fitzpatrick TB . The validity and practicality of sun-reactive skin type-I through type-vi. Arch Dermatol 1988;124:869–871. 3377516 10.1001/archderm.124.6.869

[27] von Schuckmann LA, Hughes MC, Green AC, van der Pols JC. Forearm hair density and risk of keratinocyte cancers in Australian adults. Arch Dermatol Res 2016;308:617–624. 27590883 10.1007/s00403-016-1680-5

[28] Tautan AM, Young A, Wentink E, Wieringa F. Characterization and reduction of motion artifacts in photoplethysmographic signals from a wrist-worn device. Ieee Eng Med Bio 2015;2015:6146–6149. 10.1109/EMBC.2015.7319795 26737695

[29] Boudreaux BD, Hebert EP, Hollander DB, Williams BM, Cormier CL, Naquin MR, et al Validity of wearable activity monitors during cycling and resistance exercise. Med Sci Sports Exerc 2018;50:624–633. 29189666 10.1249/MSS.0000000000001471

[30] Rainmaker DC. Polar A360 In-Depth Review. 2015. https://www.dcrainmaker.com/2015/12/polar-a360-depth-review.html.

[31] Spierer DK, Rosen Z, Litman LL, Fujii K. Validation of photoplethysmography as a method to detect heart rate during rest and exercise. J Med Eng Technol 2015;39:264–271. 26112379 10.3109/03091902.2015.1047536

[32] Vander AJ, Sherman JH, Luciano DS. Human Physiology. 3d ed. ed. New York: McGraw-Hill; 1980.

[33] Pasadyn SR, Soudan M, Gillinov M, Houghtaling P, Phelan D, Gillinov N, et al Accuracy of commercially available heart rate monitors in athletes: a prospective study. Cardiovasc Diagn The 2019;9:379–385. 10.21037/cdt.2019.06.05 PMC6732081 31555543

[34] Reiss A, Indlekofer I, Schmidt P, Van Laerhoven K. Deep PPG: large-scale heart rate estimation with convolutional neural networks. Sensors (Basel) 2019;19:20190712. 10.3390/s19143079 PMC6679242 31336894

[35] Meier M, Demirel BU, Holz C. WildPPG: A Real-World PPG Dataset of Long Continuous Recordings. arXiv preprint arXiv:241217540, 10.48550/arXiv.2412.17540, 16 December 2024, preprint: not peer reviewed.

