# Supplementary material for: Optimization and pre-use suitability selection for wrist photoplethysmography-based heart rate monitoring in patients with cardiac disease
Source: Eur Heart J Digit Health. 2025 Jul 23;6(5):1024–35. doi: 10.1093/ehjdh/ztaf084 (PMC12450509; doi:10.1093/ehjdh/ztaf084)
Supplement: ztaf084_Supplementary_Data [file ztaf084_supplementary_data.pdf]

Supplementary material

Optimisation and pre-use suitability selection for wrist photo-plethysmography based heart rate monitoring in cardiac patients

Paulien Vermunicht*, Christophe Buyck, Sebastiaan Naessens, Wendy Hens, Caro Verberckt, Emeline Van Craenenbroeck, Kris Laukens, Lien Desteghe, Hein Heidbuchel

**** Correspondence:*** *Paulien Vermunicht: Paulien.Vermunicht@uantwerpen.be*

Supplementary Figure 1. Visual representation of the baseline device guidance and the technical optimisation interventions performed on the PPG device.

Baseline

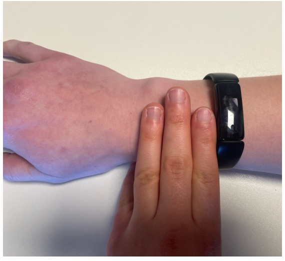

Fitbit positioned three fingers' widths above the wrist joint

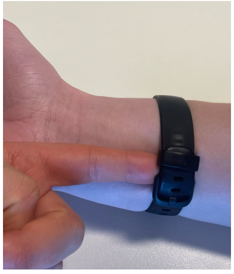

Tightened securely but comfortably

Optimisation A

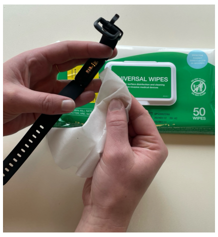

Sensor degreasing/cleaning

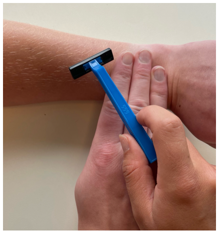

Shaving part forearm (if permission)

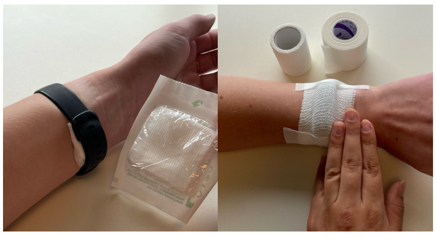

Position fixation with tape and gauze (at three fingers distance, as tight as possible)

Optimisation B

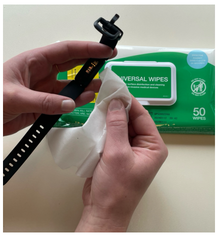

Sensor degreasing/cleaning

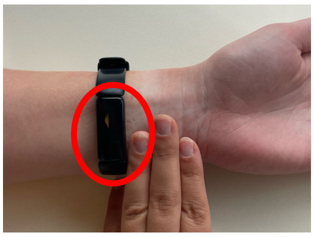

Relocated Fitbit from to the volar (bottom) wrist side

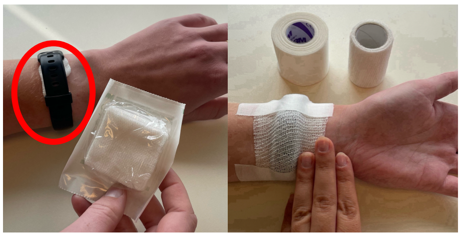

Position fixation with tape and gauze (volar side, at three fingers distance, as tight as possible)

Supplementary Figure 2. Visual representations of measured HR patterns during different wrist movements with the PPG sensor placed one and three fingers from the wrist joint for 10 individual healthy participants.

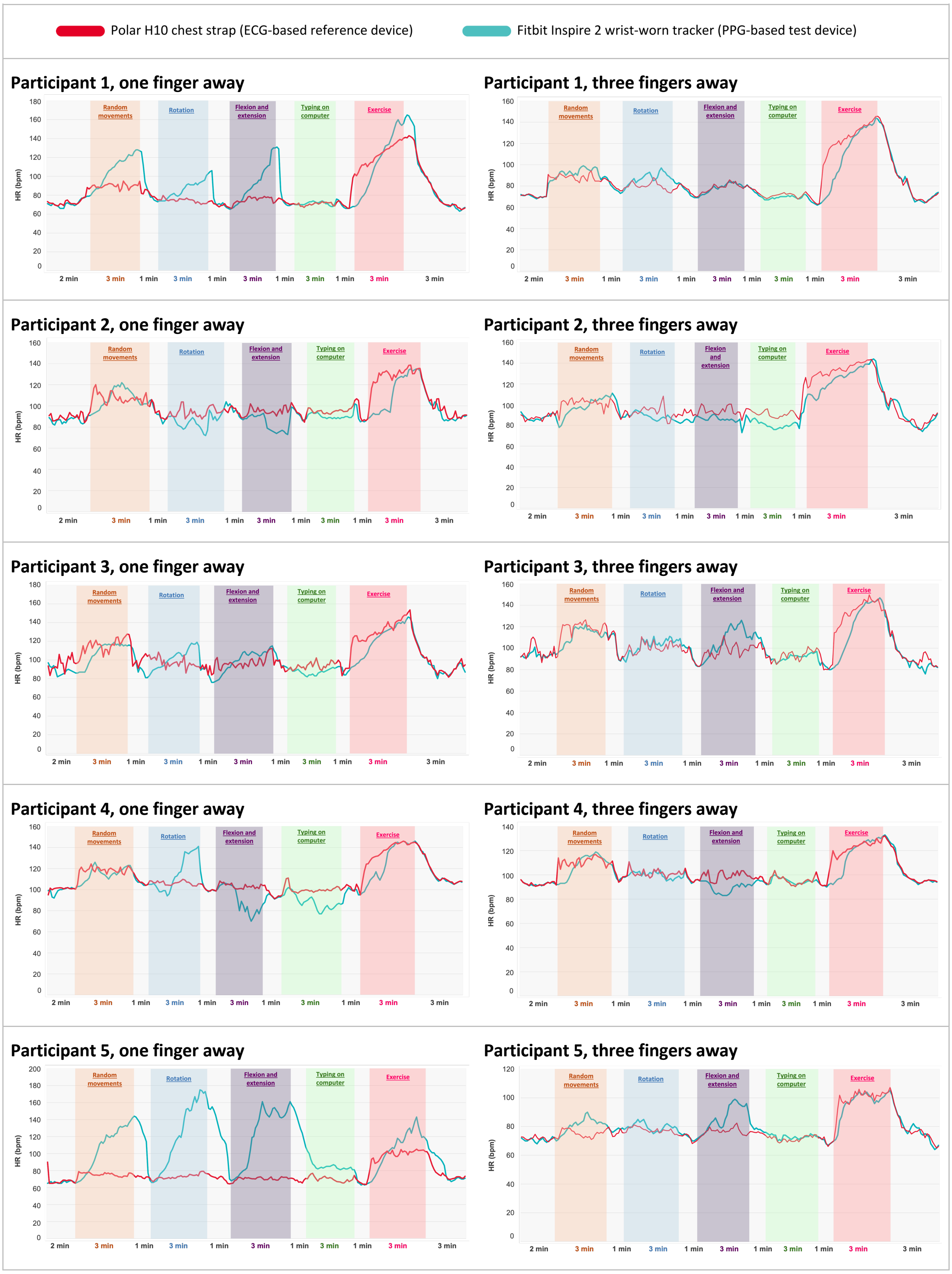

Optimisation and pre-use suitability selection for wrist photo-plethysmography based heart rate monitoring in cardiac patients
Paulien Vermunicht, Christophe Buyck, Sebastiaan Naessens, Wendy Hens, Caro Verberckt, Emeline Van Craenenbroeck, Kris Laukens, Lien Desteghe, Hein Heidbuchel

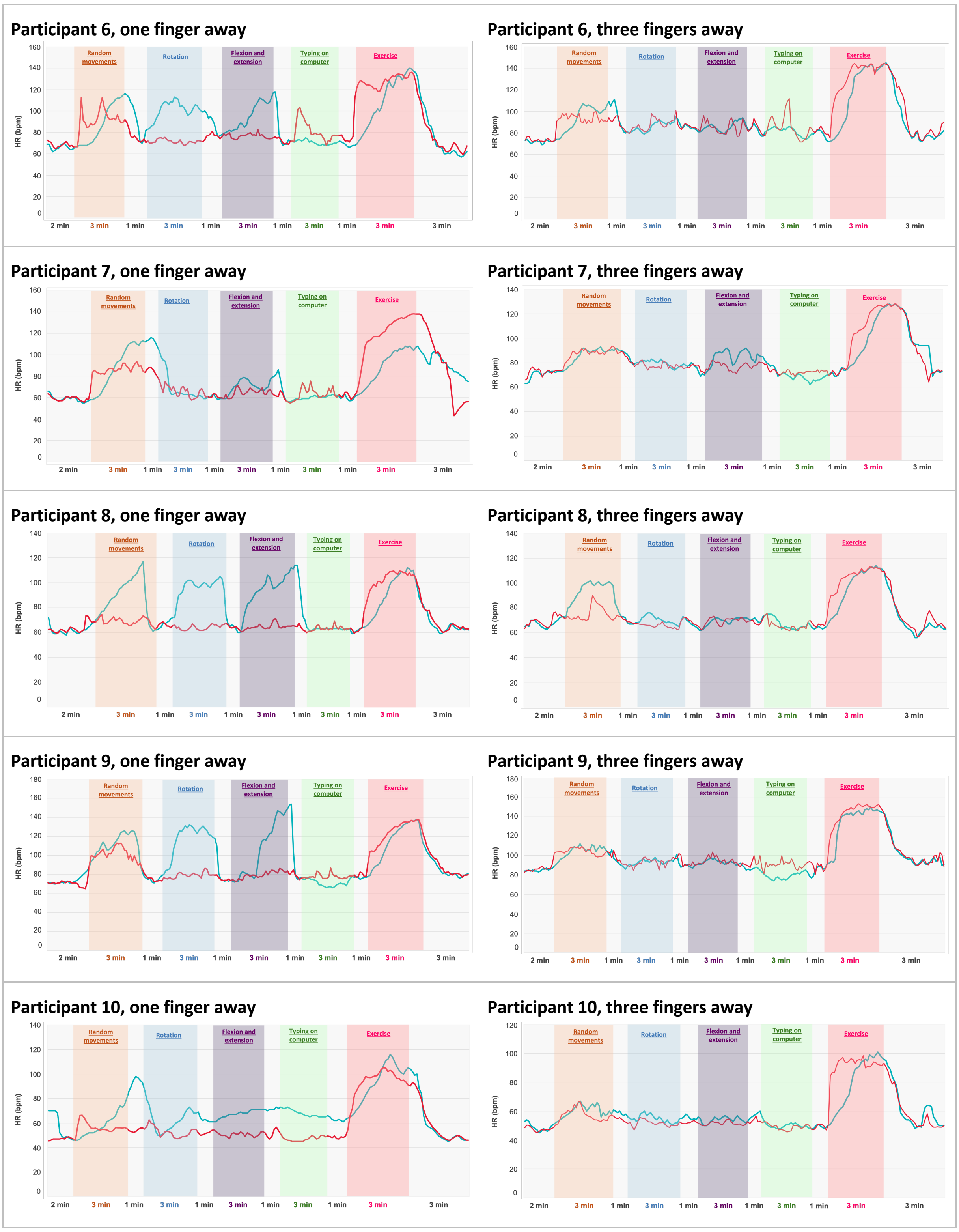

ECG: electrocardiogram; PPG: photoplethysmography; HR: heart rate; bpm: beats per minute; min: minutes.

Supplementary Figure 3. Visual representations of measured HR patterns during different sport activities for 10 individual healthy participants.

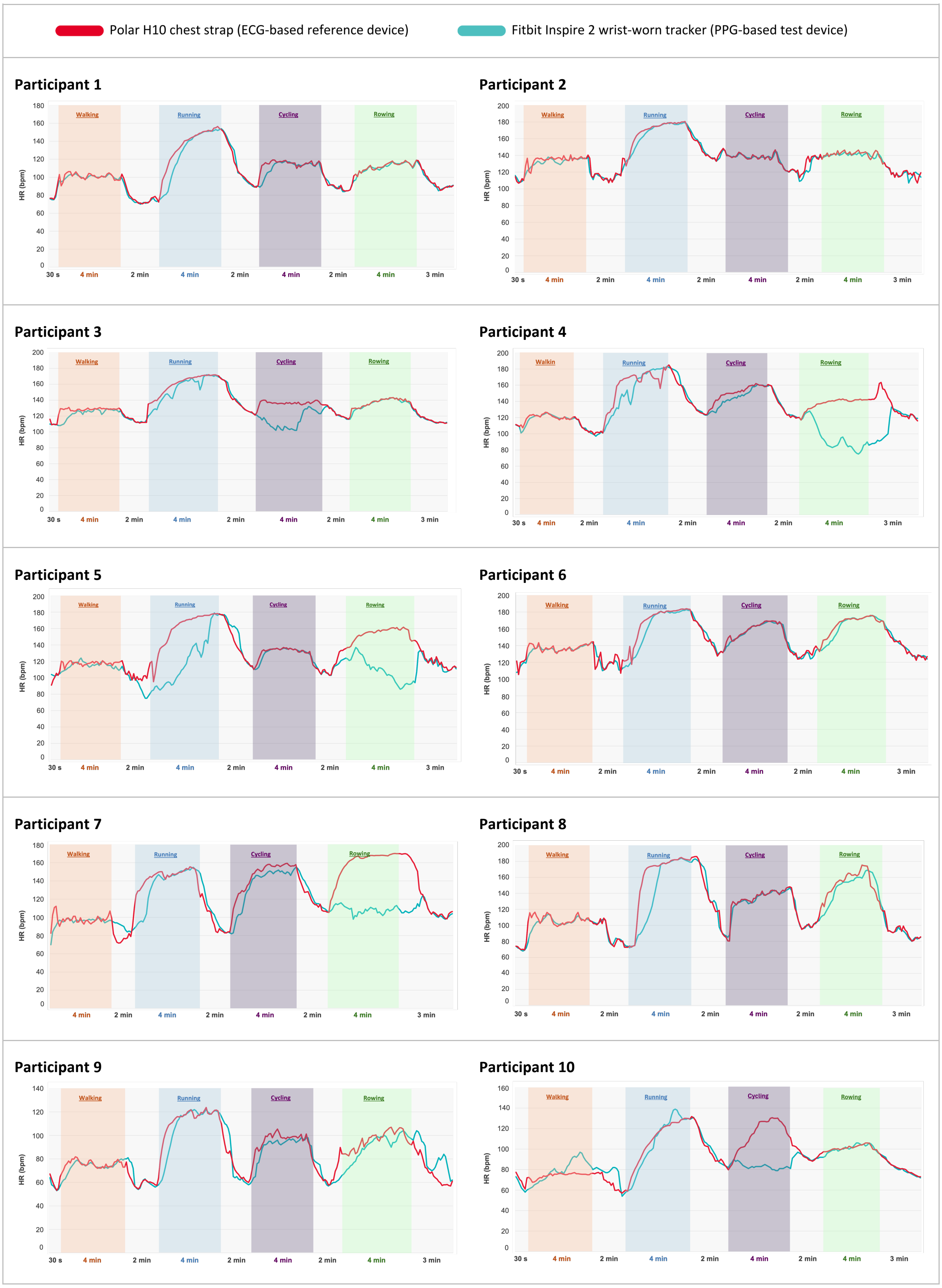

ECG: electrocardiogram; PPG: photoplethysmography; HR: heart rate; bpm: beats per minute; s: seconds; min: minutes.

Supplementary Figure 4. Percentage of accurate training time (MAPE <10%) by exercise type for (A) patients with high baseline accuracy and for (B) patients with low baseline accuracy.

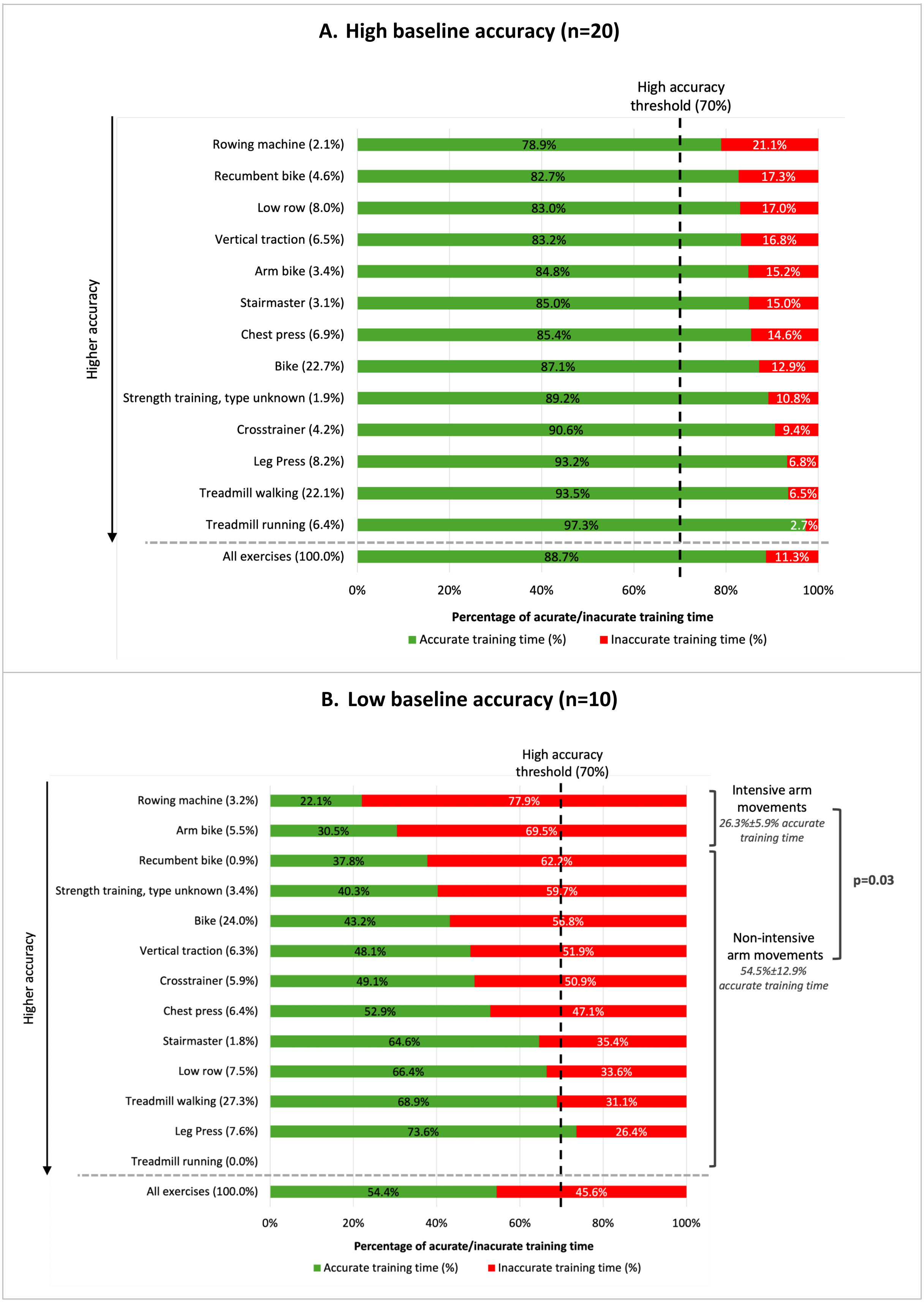

MAPE: mean absolute percentage error. Results are ranked from the lowest to the highest accuracy.
